# USP14 de-ubiquitinates vimentin and miR-320a modulates USP14 and vimentin to contribute to malignancy in gastric cancer cells

**DOI:** 10.18632/oncotarget.10706

**Published:** 2016-07-19

**Authors:** Ying Zhu, Yan Zhang, Zhenhua Sui, Yi Zhang, Min Liu, Hua Tang

**Affiliations:** ^1^ Tianjin Life Science Research Center, Department of Pathogen Biology, School of Basic Medical Sciences, Tianjin Medical University, Tianjin, 300070, China

**Keywords:** vimentin, USP14, miR-320a, gastric cancer

## Abstract

Vimentin plays important roles in the epithelial-to-mesenchymal transition (EMT). In this study, we found that vimentin was highly expressed in human gastric cancer (GC) tissues and cell lines and significantly promoted cell growth, migration and invasion. Ubiquitin-specific protease 14 (USP14) interacted with the vimentin protein, which led to its de-ubiquitination. miR-320a was found to bind to the 3′UTR of both vimentin and USP14 transcripts and downregulate the expression of both proteins. The downregulation of miR-320a upregulates vimentin expression by directly binding to the 3′UTR of vimentin to derepress expression and indirectly by augmenting USP14 to increase vimentin stability in GC cells. Taken together, these results provide new insight into malignancy in gastric cancers.

## INTRODUCTION

Gastric cancer (GC) is the fourth most frequently diagnosed malignant tumor and the third leading cause of cancer-related death worldwide as of 2012 [1, 2]. Despite recent advances in the diagnosis and treatment of GC, the mortality rate of GC remains high, and the 5-year survival rate is less than 30%. Even patients with resectable GC have a 50–90% risk of recurrence and metastasis. Therefore, it is essential to explore the molecular mechanisms of GC metastasis, which will provide important insight into new diagnostic and therapeutic approaches to improve the prognosis of GC patients.

Recent studies have shown that the conversion of epithelial cells to a mesenchymal phenotype, known as epithelial-to-mesenchymal transition (EMT), plays a key role in the migration and invasion of various cancer cells, including GC. EMT is characterized by the decreased expression of epithelial markers, such as E-cadherin, β-catenin, occludin, claudin, plakophilin, cytokeratin and desmoplakins, and the increased expression of mesenchymal markers, such as N-cadherin, vimentin and fibronectin. Currently, vimentin is attracting increased attention as a classical EMT biomarker. Vimentin, the primary intermediate filament protein of mesenchymal cells and tissues, has been found to be upregulated during EMT in epithelial cells and induces a mesenchymal phenotype and motile behavior [3].

Large quantities of evidence have shown that vimentin is upregulated in GC tissues [4], but the underlying mechanism of its upregulation is still unclear. The following factors were confirmed to modulate vimentin expression: (1) non-coding RNA in the regulation of transcription, such as miR-135a, miR-137, miR-200c, miR-141, miR-122 and long non-coding RNA [5–11]; (2) protein modification, such as phosphorylation and acetylation [12–15]. Therefore, we sought to explore whether these two mechanisms affect vimentin expression in GC, including whether other miRNAs may target vimentin and ubiquitin modification is involved in vimentin metabolism. Recent studies have suggested that ubiquitination is balanced by deubiquitination. The human genome encodes more than 100 putative deubiquitinating enzymes (DUBs), of which the primary families are the USP and UCH [16]. SiRNA interference studies on the three DUBs in cancer cells suggested that RNAi of USP14 can inhibit cellular protein degradation [17]. Studies on the expression of USP14 also assumed that USP14 was associated with cancer progression. Furthermore, high levels of USP14 expression were observed in patients with colorectal carcinoma with lymph node metastasis and liver metastasis, which indicated that USP14 may promote tumor metastasis [18, 19]. A previous study revealed that USP14 modulates cancer cell motility by deubiquitinating the chemokine receptor CXCR4 [20]. USP14 has been reported as an oncogene in different types of cancer, including GC [21]. Moreover, USP4 can modulate EMT [22]. Whether USP14 modulate the vimentin to contribute to malignancy is unclear, and we also explore the miRNAs of regulating vimentin in GC.

In this study, we examined the levels of vimentin in GC tissue samples and cell lines and evaluated the role of vimentin in the aggressiveness of GC cells. Furthermore, we explored the mechanism of the aberrant expression of vimentin and found that USP14 interacts with vimentin and de-ubiquitinates it. We also determined that USP14 and vimentin were both targets of miR-320a. Thus, miR-320a can suppress vimentin protein levels both directly through targeting its 3′UTR and indirectly through the downregulation of USP14.

## RESULTS

### Vimentin promotes the aggressiveness of gastric cancer cells

**Figure 1:**
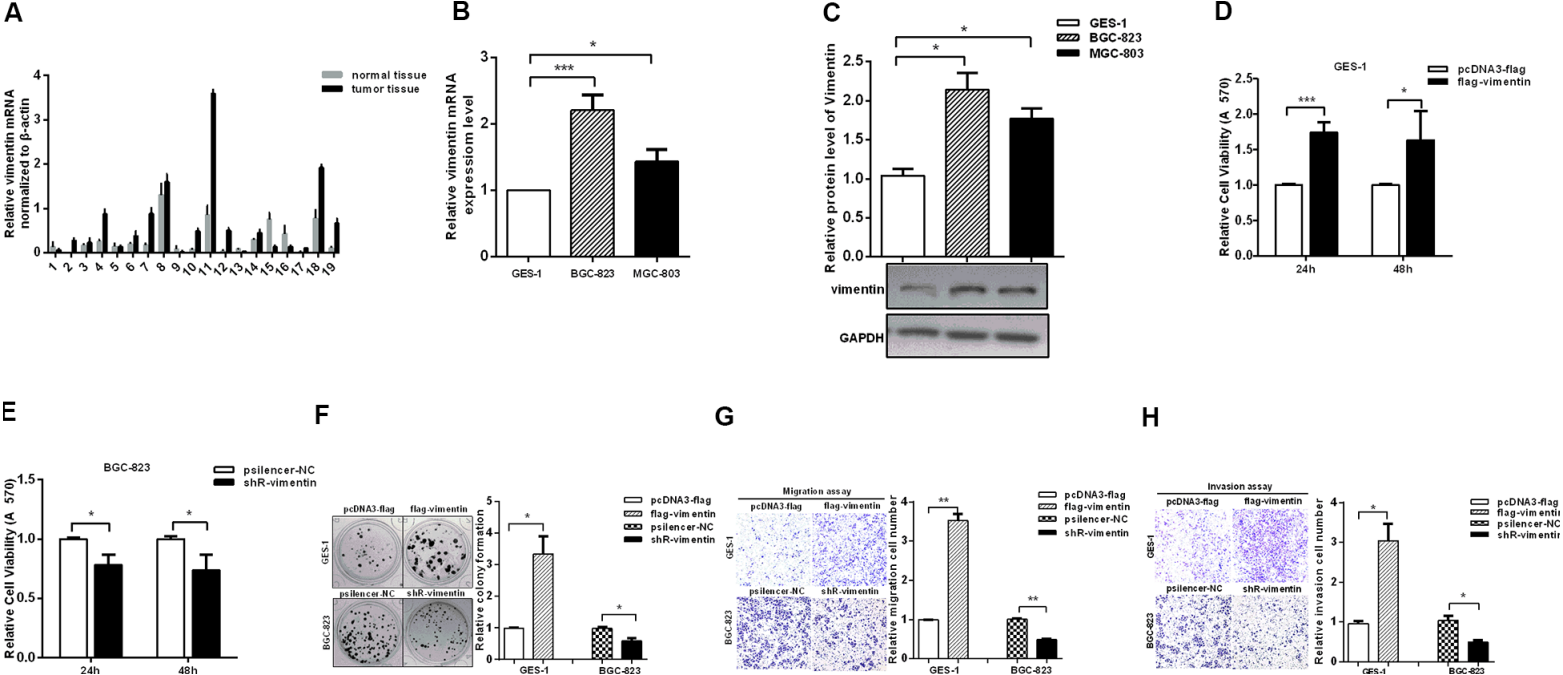
Vimentin promotes the aggressiveness of gastric cancer cells (**A** and **B**) The mRNA levels of vimentin in GC tissues (*n* = 19) and cell lines were analyzed by RT-qPCR. (**C**) Protein expression levels of vimentin in GC cell lines and the control cell line. (**D** and **E**) MTT assays performed in GES-1 cells with over-expressed vimentin and in BGC-823 cells with the inhibition of vimentin. (**F**) Colony formation assays examined the proliferation rates of GES-1 and BGC-823 transfected with flag-vimentin and shR-vimentin. (**G** and **H**) Transwell assays showed the migration and invasion in GES-1 cells with over-expressed vimentin and in BGC-823 cells with inhibited vimentin. The data are presented as the mean ± S.D. *n* = 3,*P < 0.05, **P < 0.01, ***P < 0.001 compared with the control group.

To determine the role of vimentin in the aggressiveness of GC, we investigated the expression levels of vimentin by RT-qPCR in 19 pairs of specimens of GC tissues and cell lines. The results showed that vimentin mRNA was upregulated in GC tissues compared with adjacent non-tumor tissues (Figure 1A). Similarly, the levels of vimentin mRNA in both BGC-823 and MGC-803 GC cell lines were increased compared with a normal gastric mucosa epithelial cell line (GES-1) (Figure 1B). In addition, the vimentin protein expression levels were determined by western blotting, which also showed that vimentin protein expression was increased in both BGC-823 and MGC-803 cell lines compared with GES-1 cells (Figure 1C).

Next, we detected the changes in the malignant behaviors in GES-1 cells with the overexpression of vimentin and inhibition of vimentin in GC cells. The expression plasmids and shR-vimentin are effective (Figure S1A and S1B). The results showed that the overexpression of vimentin promoted the malignant behaviors of GES-1 cells. The MTT assay demonstrated that the viabilities of GES-1 cells were increased with the overexpression of vimentin compared with the control group (Figure 1D). In contrast, the knockdown by shR-vimentin suppressed the proliferation rates of BGC-823 and MGC-803 cells (Figure 1E and Figure S1C). Colony formation assays also revealed that vimentin expression can promote the proliferation of GES-1 cells and vice versa (Figure 1F and Figure S1D). Furthermore, we utilized transwell analyses to investigate the effects of vimentin on the migration and invasion capacities. The overexpression of vimentin significantly increased migration compared with controls in GES-1 cells, whereas the inhibition of vimentin expression decreased migration approximately 50% and 40% in BGC-823 and MGC-803 cells, respectively (Figure 1G and Figure S1E). Consistently, similar results were observed in invasion assays (Figure 1H and Figure S1F). Collectively, these results indicate that vimentin is upregulated and promotes the aggressiveness of GC cells.

### USP14 can influence the expression of vimentin by affecting its ubiquitination

**Figure 2:**
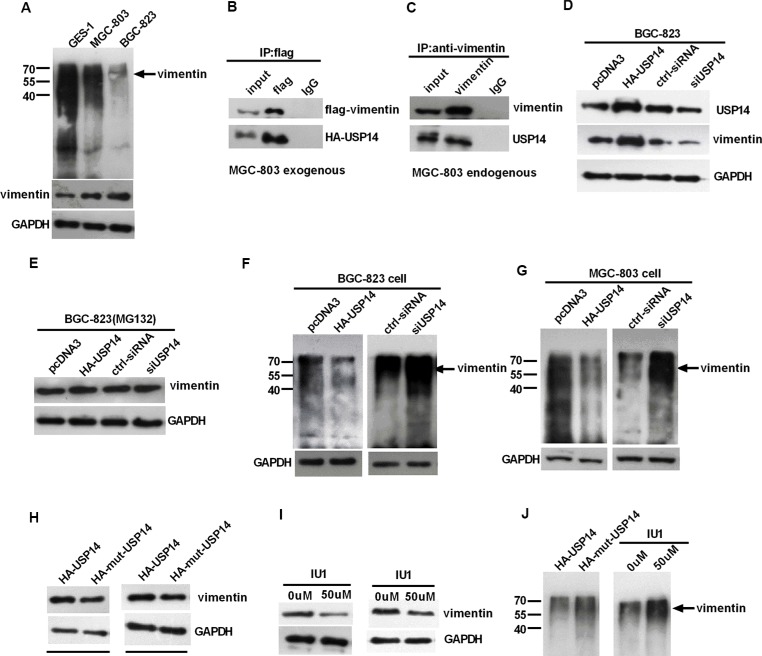
USP14 can influence the expression of vimentin by affecting its ubiquitination (**A**) Western blot detecting the ubiquitination of vimentin in GC cells and GES-1 cell lines. (**B** and **C**) Exogenous and endogenous Co-IP experiments exploring the interactions between USP14 and vimentin in MGC-803 cells. (**D** and **E**) Western blot analysis of the impact of USP14 on vimentin expression 48h after transfection, with and without the treatment of MG132 in BGC-823. (**F** and **G**) Western blot detecting the ubiquitination of vimentin in BGC-823 and MGC-803 cells after transfection with HA-USP14, siUSP14 and the respective controls. (**H**–**J**) The impact of HA-mut-USP14 and USP14 inhibitor (IU1) on vimentin protein levels and its ubiquitin levels as examined by western blotting.

The ubiquitin proteasome system is a general mechanism for endogenous protein degradation. To further explore the mechanisms of upregulation of vimentin in GC, we predicted (http://cplm.biocuckoo.org/index.php) and analyzed the ubiquitin level of vimentin in GC cells, and vimentin is also likely to be regulated via ubiquitin. We found that the ubiquitin level of vimentin decreased and USP14 increased in GC cells compared with GES-1 (Figure 2A and Figure S1G). Subsequent co-immunoprecipitation (Co-IP) experiments demonstrated an interaction between USP14 and vimentin in GC lines (Figure 2B, 2C and Figure S1H, S1I). Moreover, we observed that the increased or reduced expression of USP14 led to respective increases or decreases in the expression of vimentin (Figure 2D and Figure S1J), while the level of vimentin was not obviously changed after treatment with MG132 (Figure 2E and Figure S1K). The analysis of the ubiquitination of vimentin revealed that increased USP14 decreased the levels of ubiquitinated vimentin, and the knockdown of USP14 led to an increase in the levels of ubiquitinated vimentin (Figure 2F and 2G). Furthermore, we mutated the Cys114, His435 and Asp451 of USP14 to Ala to eliminate its enzyme activities [23, 24] and re-evaluated the vimentin protein levels and the ubiquitination levels. After eliminating the USP14 enzyme activity, compared with HA-USP14, the vimentin protein level was reduced and the ubiquitination levels were increased, which was further verified using IU1 (USP14 inhibitor) (Figure 2H–2J and Figure S1L).

### USP14 increases the malignant behavior of gastric cancer cells

**Figure 3:**
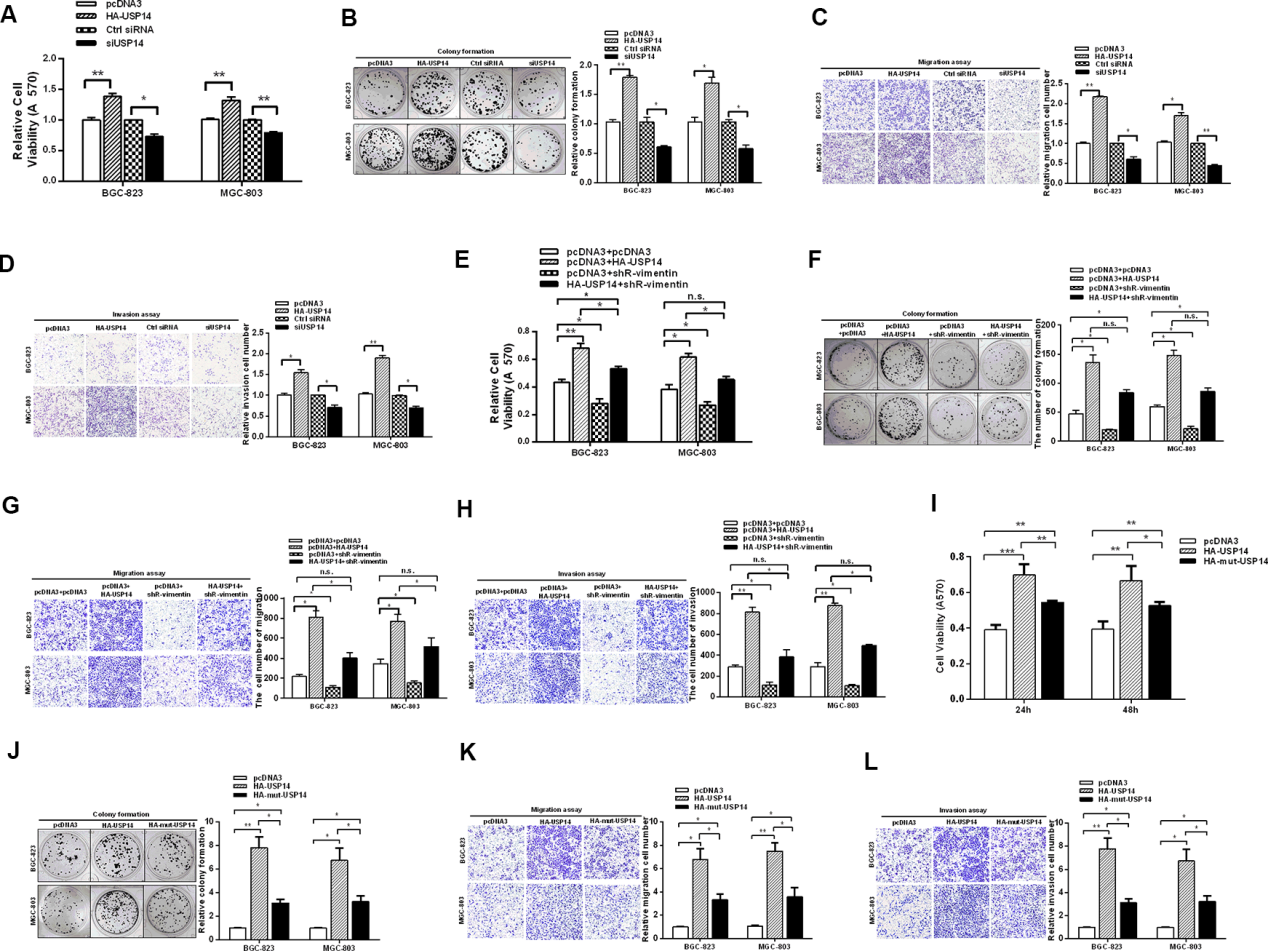
USP14 increases the malignant behavior of gastric cancer cells (**A**) An MTT assay tested the viabilities of BGC-823 and MGC-803 cells transfected with HA-USP14 and siUSP14. (**B**) Colony formation, (**C**) migration and (**D**) invasion of BGC-823 and MGC-803 cells were affected by transfection with HA-USP14 and siUSP14. (**E**–**H**) MTT, colony formation, migration and invasion assays were performed to investigate the effects of shR-vimentin on USP14's promotion of gastric malignant behaviors. (**I**–**L**) The biological functions of mut-USP14 and WT-USP14 in GC cells, as determined by MTT, colony formation, migration and invasion assays. The data are presented as the mean ± S.D. *n* = 3, *P < 0.05, **P < 0.01 compared with the control group.

According to the above results, USP14 de-ubiquitinates vimentin and increases its expression levels, which may influence the aggressiveness of GC cells. To further study the roles of USP14 in GC, we employed MTT and colony formation assays to analyze the effect of USP14 on cell viability and growth capacity. The MTT assay showed that USP14 overexpression increased the cell viability in BGC-823 and MGC-803 cells, and the knockdown of USP14 repressed the cell viability in BGC-823 and MGC-803 cells (Figure 3A). Similarly, the rate of colony formation was increased by USP14 overexpression but was reduced by siUSP14 (Figure 3B). Furthermore, the ectopic expression of USP14 increased migration in BGC-823 and MGC-803 cells, respectively, whereas knockdown of USP14 led to approximately 0.4-fold and 0.5-fold decreases in migration (Figure 3C). Similarly, HA-USP14 promoted invasive activity in the GC cell lines (Figure 3D). Taken together, these results suggest that USP14 accelerates the malignant behavior of GC cells.

In addition, complementary function assays between USP14 and vimentin were performed. We did not observe that shR-vimentin could completely offset the effect of USP14 in promoting GC malignant behavior (Figure 3E–3H). Accordingly, we detected the functional assays of biological functions of mut-USP14 in GC cells. The results showed that the mut-USP14 still had a partial effect in promoting the malignant behavior of GC (Figure 3I–3L), which may be because USP14 acts on proteasomes and may also affect the signaling pathways of the cancer cells [25, 26].

### USP14 and vimentin are suppressed by miR-320a

**Figure 4:**
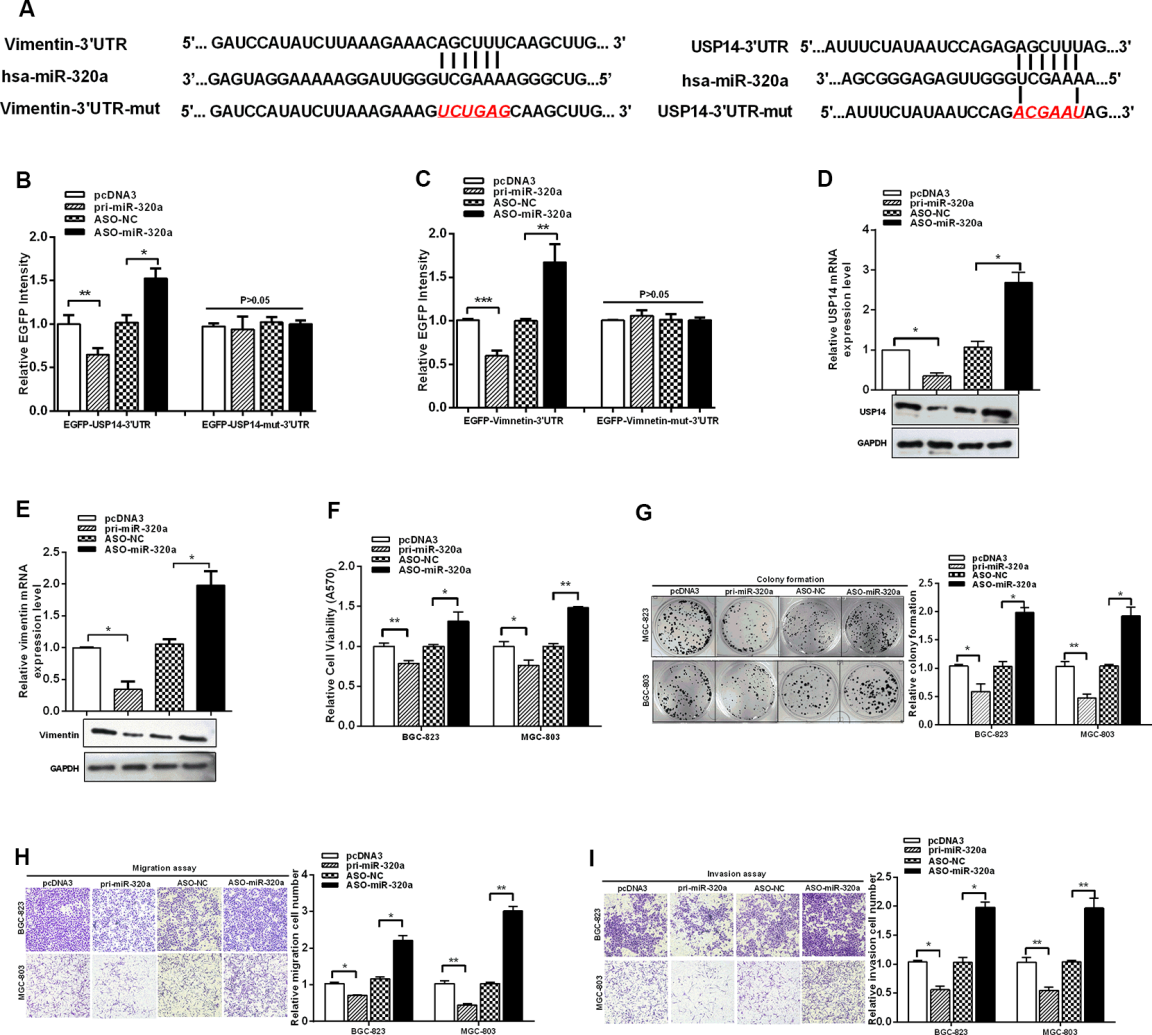
USP14 and vimentin are suppressed by miR-320a (**A**) The vimentin and USP14 3′UTR and mutant 3′UTR containing a miR-320a-binding sites are shown. (**B** and **C**) BGC-823 cells were transfected with EGFP reporter vectors containing either wild-type or mutant USP14 3′UTR and wild-type or mutant vimentin 3′UTR. The relative EGFP intensity was determined after transfection. (**D** and **E**) mRNA and protein expression levels of USP14 and vimentin were measured by RT-qPCR and western blotting after transfection with pri-miR-320a and ASO-miR-320a. (**F**) MTT, (**G**) colony formation, (**H**) migration and (**I**) invasion of BGC-823 and MGC-803 cells assays were affected by transfection with pri-miR-320a and ASO-miR-320a. The data are presented as the mean ± S.D. *n* = 3, *P < 0.05, **P < 0.01, ***P < 0.001 compared with the control group.

Our experiments determined that USP14 influenced the protein levels of vimentin by de-ubiquitination. However, we found that vimentin mRNA levels were also increased in GC tissues. To investigate the increase in vimentin at the transcriptional level, we used bioinformatics to predict the upstream candidate miRNAs (http://www.mirbase.org/; http://www.targetscan.org/). In comparing the functions of these miRNAs, miR-320a was chosen for further analysis because it has been identified to be decreased in GC and its expression in serum has been related to GC [27, 28]. Surprisingly, bioinformatics showed that miR-320a could target vimentin as well as directly bind USP14, which might contribute to the tumor suppressor role of miR-320a in GC (Figure 4A).

Because the 3′ UTR of both USP14 and vimentin contain binding sites for miR-320a, we investigated whether USP14 and vimentin are direct targets of miR-320a in GC cells. We generated EGFP reporters containing the USP14 3′UTR or the vimentin 3′UTR, with either the wild-type sequence or mutations in the miR-320a binding sequence (Figure 4A). Reporter assays demonstrated that miR-320a overexpression decreased the fluorescence intensities of the wild-type reporters in BGC-823 cells, while ASO-miR-320a expression increased the fluorescence intensities of the wild-type reporters in BGC-823 cells; miR-320a overexpression did not influence the fluorescence intensities of the mutant reporters (Figure 4B and 4C). Consistent with these findings, RT-qPCR and western blotting showed that miR-320a reduced the mRNA and protein expression of USP14 and vimentin (Figure 4D–4E and Figure S2C, S2D). These data indicate that miR-320a targets and downregulates the expression of USP14 and vimentin.

The above data demonstrate that both USP14 and vimentin promoted malignancy and were downregulated by miR-320a in GC cells. Next, to examine the influence of miR-320a on malignancy in GC cells, we constructed a miR-320a plasmid, synthesized ASO-miR-320a (2′-O-methyl-modified antisense oligonucleotide of miR-320a) and verified the effectiveness of pi-miR-320a and ASO-miR-320a (Figure S2A and S2B). Subsequently, MTT, colony formation, migration and invasion assays were performed to explore the functional roles of miR-320a in GC cells. We found that miR-320a functions as a tumor suppressor in GC cells by suppressing cell proliferation, migration and invasion (Figure 4F–4I). Together, these results indicate that miR-320a downregulates USP14 and vimentin and functions as a tumor suppressor in GC cells.

### miR-320a functions as a tumor suppressor in GC cells via USP14 and vimentin

**Figure 5:**
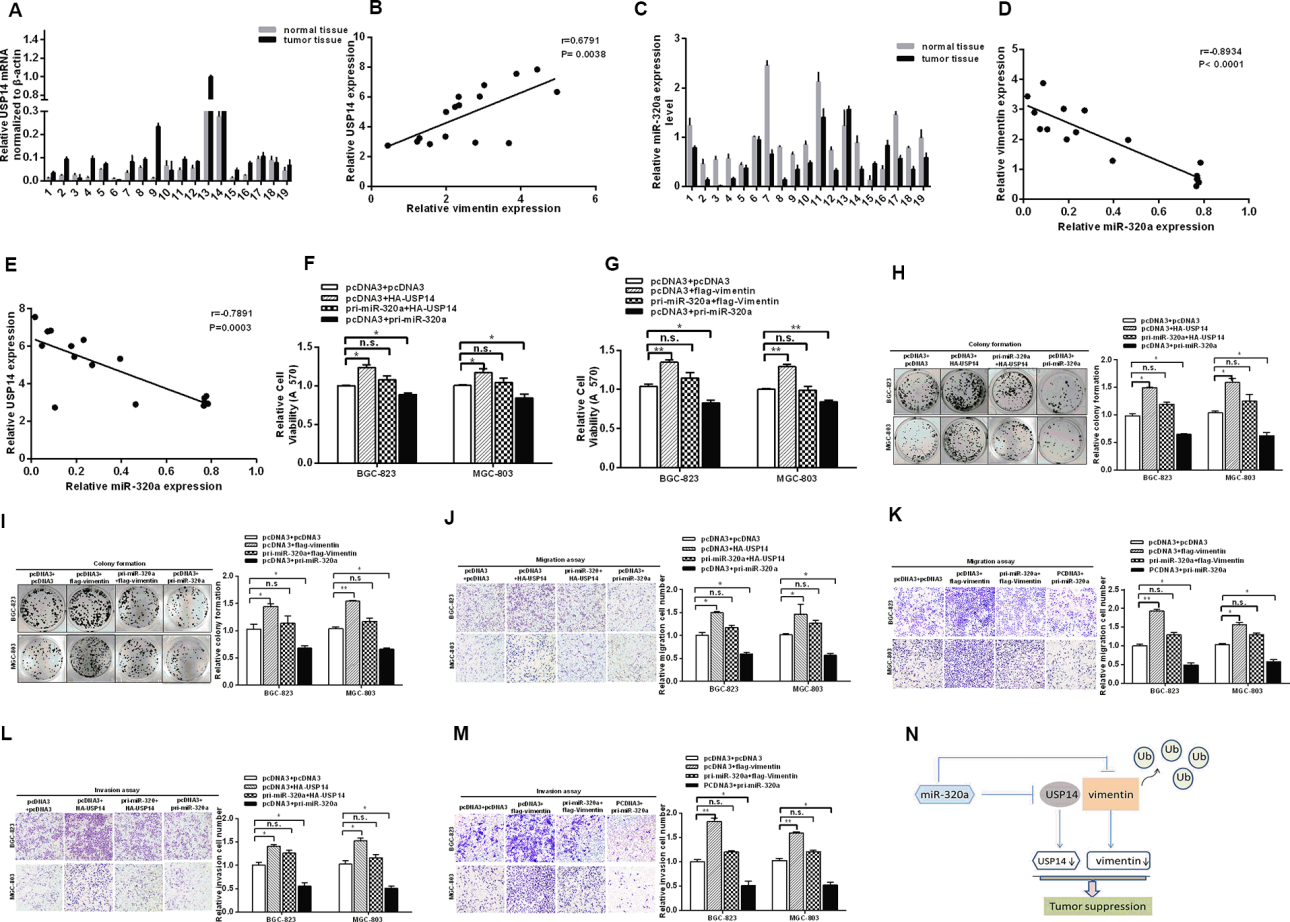
miR-320a functions as a tumor suppressor in GC cells via USP14 and vimentin (**A**) The mRNA levels of USP14 in GC tissues were analyzed by RT-qPCR. (**B**) The positive correlation of USP14 and vimentin in GC tissues. (**C**) The mRNA levels of miR-320a in GC tissues analyzed by RT-qPCR. (**D** and **E**) Pearson's correlation analysis between miR-320a/USP14 and miR-320a/vimentin levels in GC tissues (*n* = 19). Overexpression of USP14 and vimentin rescues the effect of miR-320a on cell viability (**F** and **G**), colony formation (**H** and **I**), migration (**J** and **K**) and invasion (**L** and **M**) in BGC-823 and MGC-803 cells, respectively. The data are presented as the mean ± S.D. *n* = 3, *P < 0.05, **P < 0.01 compared with the control group. (N) miR-320a not only suppresses vimentin directly but also binds to USP14 to inhibit vimentin indirectly in GC.

We examined the expression levels of vimentin and USP14 in our GC sample cohorts by RT-qPCR and found higher expression levels in cancer compared with the paired adjacent normal tissues (Figures 1A and 5A). The GC cells yielded similar results (Figure 1B and Figure S2E), which were consistent with previous studies [4, 21, 22, 29–33]. The correlation analysis between USP14 and vimentin in GC tissues revealed their positive correlations (Figure 5B). In addition, we also examined the expression levels of miR-320a in clinical GC specimens and GC cells by RT-qPCR (Figure 5C and Figure S2F) and analyzed the correlation between vimentin/USP14 and miR-320a expression. As shown in Figure 5D and 5E, miR-320a expression was inversely correlated with the expressions of vimentin and USP14 in GC tissues.

To confirm that the tumor suppressor role of miR-320a in GC is mediated by the repression of the expression of vimentin and USP14, rescue experiments were performed. The promoting effects of USP14 and vimentin on MTT and the rate of colony formation were rescued when a miR-320a expression vector was co-transfected (Figure 5F–5I). Furthermore, the suppression of migration and invasion induced by miR-320a was abrogated under USP14 and vimentin overexpression (Figure 5J–5M). Notably, the abrogating effects of USP14 on invasion and migration were less than those of vimentin, which may be due to the direct inhibition of vimentin by miR-320a. In short, these results suggest that the tumor suppressor role of miR-320a in GC cells is mediated or at least partially mediated by the downregulation of USP14 and vimentin expression.

## DISCUSSION

The epithelial-to-mesenchymal transition (EMT) is one of the main molecular mechanisms involved in oncogenesis to promote cancer progression [34]. The loss of E-cadherin expression and the gain of vimentin expression are thought to be the most important molecular markers of EMT [35, 36]. However, vimentin also plays crucial roles in a number of biological processes. For example, vimentin is involved in the regulation of mitochondrial motility and membrane potential by Rac1 [37], and vimentin forms a complex with 14-3-3 and beclin 1 to inhibit autophagy via an AKT-dependent mechanism in tumorigenesis [38]. In recent years, regulatory factors of vimentin have been identified. For example, alpha-catulin colocalizes with vimentin intermediate filaments and functions in pulmonary vascular endothelial cell migration via ROCK [39], and proteomic analysis revealed the important role of vimentin in human HeLa cells [40]. Here, we found that vimentin was upregulated in human GC tissues and cell lines, and the upregulation of vimentin promoted the aggressiveness of GC cells.

The ubiquitin-proteasome system (UPS) plays an important role in maintaining cellular protein homeostasis and is associated with a variety of human diseases, including cancer and various diseases [18, 19, 41]. The ubiquitin-proteasome system is the major pathway of non-lysosomal endogenous protein degradation, which plays an important role in various cellular responses, including cell division, proliferation, and apoptosis [42]. This fundamental role of the proteasome singles it out as a unique target for anticancer therapy [43]. Previous research has shown that USP14 expression is associated with leukemia [44], colorectal cancer [18], intrahepatic cholangiocarcinoma [45], and lung carcinoma [32] and that USP14 inhibits Dengue virus replication [46]. However, to the best of our knowledge, no report has demonstrated the function and mechanism of USP14 in GC cells. In this study, we further verified the high expression levels of USP14 in GC cell lines and tissues and found that USP14 interacts with vimentin, de-ubiquitinates it, and increases its expression level in GC cells, which promotes cell aggressiveness, including the growth, migration and invasion of gastric cancer cells.

Studies have shown that the regulation of vimentin may occur at the protein and mRNA levels. Some non-coding RNAs could regulate the transcription of vimentin. For example, miR-138 modulates metastasis and EMT in breast cancer cells by targeting vimentin [7], miR-122 regulates hypoxia-inducible factor-1 and vimentin in hepatocytes [47], miR-141 downregulates the expression of vimentin in renal tubular epithelial cells [48], and miR-200c suppresses vimentin [49, 50]. However, protein phosphorylation and acetylation can also affect the expression of vimentin [12–15]. In our study, we explored the two mechanisms of upregulation of vimentin at the protein and mRNA levels in GC. We found that both USP14 and vimentin were direct targets of miR-320a, which regulate dual target functions.

The dysregulated expression of miRNAs has been well demonstrated in nearly all types of human cancers, and numerous miRNAs are involved in the formation and progression of tumors by regulating oncogenes and tumor suppressor genes. In our study, we found that USP14 and vimentin are target genes of miR-320a. The restoration of miR-320a expression can suppress GC cell proliferation, migration and invasion by targeting both USP14 and vimentin, which shows that miR-320a acts as a tumor suppressor in GC. Recent reports support this notion. For example, miR-320a has been found to be involved in pathogenesis in colorectal cancer [51], breast cancer [52], esophageal cancer [53], hepatoma [54] and chronic myeloid leukemia [55]. Furthermore, miR-320a inhibits the metastasis of salivary adenoid cystic carcinoma [56].

In summary, our results demonstrated that vimentin in human GC tissues and cell lines was upregulated due to its de-ubiquitination after interactions with USP14 and miR-320a, which could promote the aggressiveness of GC cells. Both USP14 and vimentin are the direct targets of miR-320a, which suppresses GC cell proliferation, migration and invasion. Thus, miR-320a not only suppresses vimentin directly but also binds to USP14 to inhibit vimentin indirectly in GC (Figure 5N). These findings may provide new insights into the mechanisms of malignancy in gastric cancer.

## MATERIALS AND METHODS

### Human GC tissue samples

Nineteen gastric cancer tissue samples and matched adjacent normal tissue samples were obtained from Tianjin Cancer Hospital, Tianjin Medical University, in accordance with the ethical standards of the institutional committee. The diagnosis of gastric carcinoma was confirmed by pathological examination.

### miRNA target prediction

The putative miRNA targets were predicted by TargetScan and miRBase.

### Plasmid constructs and oligos

The fragment (82 bp) containing the miR-320a precursor sequence was amplified from gastric cancer cell genomic DNA and cloned into the pcDNA3 vector between the BamHI and EcoRI sites. The USP14 and vimentin sequences were amplified from the cDNA of GC cells by PCR and cloned into flag/pcDNA3 vectors between the EcoRI and Xbal sites. The shRNA vector against vimentin was constructed by annealing synthesized oligos and ligating them into psilencer 2.1-U6 neo between the HindIII and BamHI sites. Antisense oligos against miR-320a (ASO-miR-320a) and siRNA for USP14 were chemically modified and synthesized at GenePharma, Inc. (Shanghai, China). The 3′-UTR fragments of the USP14 and vimentin genes containing the putative miR-320a binding sites and the respective mutated binding sites were inserted into pcDNA3-EGFP vectors between the BamHI and EcoRI sites by oligo annealing and ligation. The mutations (Cys114, His435 and Asp451) of HA-USP14 plasmids were constructed with PCR-based site-directed mutagenesis using the QuikChange Site-Directed Mutagenesis Kit (Stratagene, CA). All the primers and oligos used in this study are listed in Table 1.

**Table 1:** The primers and oligonucleotides used in this work

Name	Sequence (5′-3′)
pri-miR-320a-sense	5′-GTTGGATCCGGCGTTTCCTTCCGACATG-3′
pri-miR-320a-antisense	5′-GCTGAATTCGTCCACTGCGGCTGTTCC-3′
ASO-miR-320a	5′-UCGCCCUCUCAACCCAGCUUUU-3′
ASO-NC	5′-CAGUACUUUGUGUAGUACAA-3′
USP14-siRNA-sense	5′-UCAGCAUCGUAACACCAGAAGAUAU-3′
USP14-siRNA-antisense	5′-AUCUUCUGGUGUUACGAUGCUGACA-3′
Ctrl-siRNA-sense	5′- UUCUCCGAACGUGUCACGUTT-3′
Ctrl-siRNA-antisense	5′-ACGUGACACGUUCGGAGAATT-3′
USP14-UTR-sense	5′-GATCCATTTCTATAATCCAGAGCTTTAGAAGCTTG-3′
USP14-UTR-antisense	5′-AATTCAAGCTTCTAAAGCTCTGGATTATAGAAATG-3′
USP14-UTR-mut-sense	5′-GATCCATTTCTATAATCCAGACGAATAGAAGCTTG-3′
USP14-UTR-mut-antisense	5′-AATTCAAGCTTCTATTCGTCTGGATTATAGAAATG-3′
USP14-qPCR-sense	5′-TGATGTGATGCAATCTGTG-3′
USP14-qPCR-antisense	5′-ATCCTGCCCATTCTATTC-3′
Vimentin-sense	5′-CCGAATTCATGTCCACCAGGTCCGTG-3′
Vimentin-antisense	5′-GTGCTCTAGAGCTTCAAGGTCATCGTGATG-3′
vimentin-qPCR-sense	5′-GGACCAGCTAACCAACGACA-3′
vimentin-qPCR-antisense	5′-AAGGTCAAGACGTGCCAGAG-3′
vimentin-UTR-sense	5′-GATCCATATCTTAAAGAAACAGCTTTCAAGCTTG-3′
vimentin-UTR–antisense	5′-AATTCAAGCTTGAAAGCTGTTTCTTTAAGATATG-3′
vimentin-UTR-mut-sense	5′-GATCCATATCTTAAAGAAAGTCTGAGCAAGCTTG-3′
vimentin-UTR-mutantisense	5′-AATTCAAGCTTGCTCAGACTTTCTTTAAGATATG-3′
shR-vimentin-sense	5′-GATCCCAGGATGAGATTCAGAATATGCTCGAGCATATTCTG AATCTCATCCTGTTTTTGA-3′
shR-vimentin-antisense	5′-AGCTTCAAAAACAGGATGAGATTCAGAATATGCTCGAGCA TATTCTGAATCTCATCCTGG-3′
β-actin-sense	5′-CGTGACATTAAGGAGAAGCTG-3′
β-actin-antisense	5′-CTAGAAGCATTTGCGGTGGAC-3′
USP14-114-mut-sense	5′-GGTA ACACTGCTTA CATGAATGCC ACAGTTCAG-3′
USP14-114-mut-antisense	5′-CATTCATGTAAGCAGTGTTACCAAGGTTTGTC-3′
USP14-435-mut-sense	5′-GTTCTTCAGGTGCTTATGTATCATGGGTG-3′
USP14-435-mut-antisens	5′-ATACATAAGCACCTGAAGAACTAGACCTTC-3′
USP14-451-mut-sense	5′-TAAGTTTGCTGATGACAAAGTCAGCATCG-3′
USP14-451-mut-antisense	5′-CTTTGTCATCAGCAAACTTAATCCATTCATC-3′

### Cell culture and transfection

The gastric cancer cell line BGC-823 was cultured in RPMI1640, and the MGC-803 gastric cancer cell line and the normal gastric mucosa epithelial cell line GES-1 were cultured in DMEM (GIBCO BRL, Grand Island, NY) supplemented with 10% fetal bovine serum (FBS), 100 IU/mL of penicillin, and 100 μg/mL streptomycin and were incubated at 37°C with 5% CO_2_. Transfections were performed using Lipofectamine 2000 reagent (Invitrogen, Carlsbad, CA) according to the manufacturer's protocol.

### EGFP fluorescent reporter assay

BGC-823 cells or MGC-803 cells were co-transfected with pcDNA3, pri-miR-320a, ASO-NC and ASO-miR-320a in a 48-well plate followed by pcDNA3/EGFP-USP14/vimentin-3′UTR or pcDNA3/EGFP-USP14/vimentin mut-3′UTR. pcDNA3 or ASO-NC was used as the control. A separate RFP expression vector (pDsRed2-N1; Clontech, Mountain View, CA) was used for normalization. The cells were collected and lysed at 48 h post-transfection. The EGFP and RFP fluorescence intensities were detected with the F-4500 Fluorescence Spectrophotometer (Hitachi, Tokyo, Japan).

### RNA preparation and quantitative RT-PCR

Large and small RNAs from tissues were isolated using the mirVana miRNA Isolation Kit (Ambion, Austin, TX) according to the manufacturer's protocol. Quantitative RT-PCR (qRT-PCR) was performed to detect the relative transcript levels of miR-320a, USP14 and vimentin. PCR was performed under the following conditions: 94°C for 4 min followed by 40 cycles of 94°C for 1 min, 56°C for 1 min and 72°C for 1 min. The relative expression levels of the gene of interest were calculated by the 2ΔΔCt method. All primers were synthesized by AuGCT Inc. (Beijing, China).

### Western blotting

Cultured cells were lysed in RIPA buffer, and their lysates were analyzed by a standard western blot procedure. Glyceraldehyde-3-phosphate dehydrogenase (GAPDH) was used as an endogenous normalizer. The specific antibodies were obtained from Saierbio (Tianjin, China).

### Cell viability assay

Cells were seeded 24 h after transfection in 96-well plates at 3,000 cells per well. The 3-(4,5-dimethylthiazol-2-yl)-2,5-diphenyl-tetrazolium bromide (MTT) assay was used to determine cell viability at 24, 48, and 72 h after the cells were seeded. The absorbance at 570 nm was measured using an uQuant Universal Microplate Spectrophotometer (BioTek, Winooski, VT).

### Colony formation assay

For the colony formation assay, the number of viable cell colonies was determined after 15 days (BGC-823 and MGC-803 cells) after the inoculation of 300 cells/well in triplicate in 12-well plates. The cells were stained with crystal violet. The ability to form colonies was evaluated by determining the colony formation number.

### *In vitro* migration and invasion assays

BGC-823 and MGC-803 cells were transfected with pri-miR-320a, ASO-miR-320a, HA-USP14, flag-vimentin or the corresponding controls. A 24-well Boyden chamber with an 8-nm pore size polycarbonate membrane (Millipore, Merck KGaA, Darmstadt, Germany) was used to analyze the migration and invasion of the tumor cells. For the invasion assay, the membrane was coated with Matrigel (Clontech). Cells in three different fields of view at 100× magnification were counted, and the measurement was expressed as the average number of cells per field of view. All assays were performed in triplicate.

### *In vitro* ubiquitination assay

BGC-823 and MGC-803 cells grown in 30 cm^2^ cell culture flasks were transiently transfected with the plasmids pcDNA, HA-USP14, ctrl-siRNA or siUSP14 and cotransfected with flag-vimentin (endogenous ubiquitination without cotransfection of flag-vimentin) and harvested 48 h post-transfection. The transfectants were treated with 20 μM MG132 for 8 h before harvest. The cells were lysed in a lysis buffer (25 mM Tris-Cl PH 7.4, 150 mM NaCl, 1% NP-40, 10% glycerol, 1 mM DTT, 10 mM NaF, 20 mM sodium vanadate, 8 mM β-glycerophosphate, 50 mM chloroacetamide and protease inhibitor cocktail) at 4°C for 30 min. After centrifugation, the supernatant was incubated with anti-Flag (endogenous ubiquitination incubated with anti-vimentin) and incubated for 16h on a rotating wheel at 4°C. The protein-antibody complexes were precipitated by the lysate with BioepitopeR protein G/A agarose IP reagent beads (Bioworld Technology, Louis, CA) and incubated on a rotating wheel for 12 h at 4°C. After incubation, the beads were then washed with ubiquitination buffer four times. The agarose beads bound by antibody and investigative proteins were mixed with SDS-PAGE sample buffer and separated in 10% SDS-PAGE gels, immunoprecipitates were loaded into polyacrylamide gels and transferred to a polyvinylidene fluoride (PVDF) membrane (Millipore, Billerica, MA, USA). Immunoblotting was carried out using anti-ubiquitin antibody (Sigma). Endogenous ubiquitination used anti-ubiquitin antibody and the second antibody Fab-HRP.

### Co-immunoprecipitation

BGC-823 and MGC-803 were transfected with flag-vimentin and HA-USP14 as described above. At 48 h post-transfection, GC cells were lysed with buffer containing 20 mM imidazole, 300 mM KCl, 5 mM MgCl_2_, 1% TritonX-100, 5% glycerol and complete protease inhibitor cocktail (Roche) at 4°C for 30 min. After centrifugation, 1 ml of the resulting supernatant was incubated with anti-Flag antibodies (endogenous CoIP incubated with anti-vimentin) at 4°C for 16 h. The protein-antibody complexes were precipitated by incubating the lysates with Bioepitope R protein G/A agarose IP reagent beads (Bioworld Technology, Louis, CA) for 6 h under constant rotation at 4°C. After incubation, the resin was washed four times with lysis buffer, and proteins were eluted with 2× loading buffer, boiled for 5 min, and processed for Western blotting.

### Statistical analysis

Statistical significance was determined using Student's *t*-test. The data are expressed as the means ± S.D. A *P*-value less than 0.05 was considered statistically significant. All experiments were repeated more than three times, and each experiment was performed using three wells per condition.

## SUPPLEMENTARY FIGURES


