# Supplementary material for: USP14 de-ubiquitinates vimentin and miR-320a modulates USP14 and vimentin to contribute to malignancy in gastric cancer cells
Source: Oncotarget. 2016 Jul 19;8(30):48725–36. doi: 10.18632/oncotarget.10706 (PMC5564720; doi:10.18632/oncotarget.10706)
Supplement: Supplementary file 1 [file oncotarget-08-48725-s001.pdf]

USP14 de-ubiquitinates vimentin and miR-320a modulates USP14 and vimentin to contribute to malignancy in gastric cancer cells

Supplementary Materials

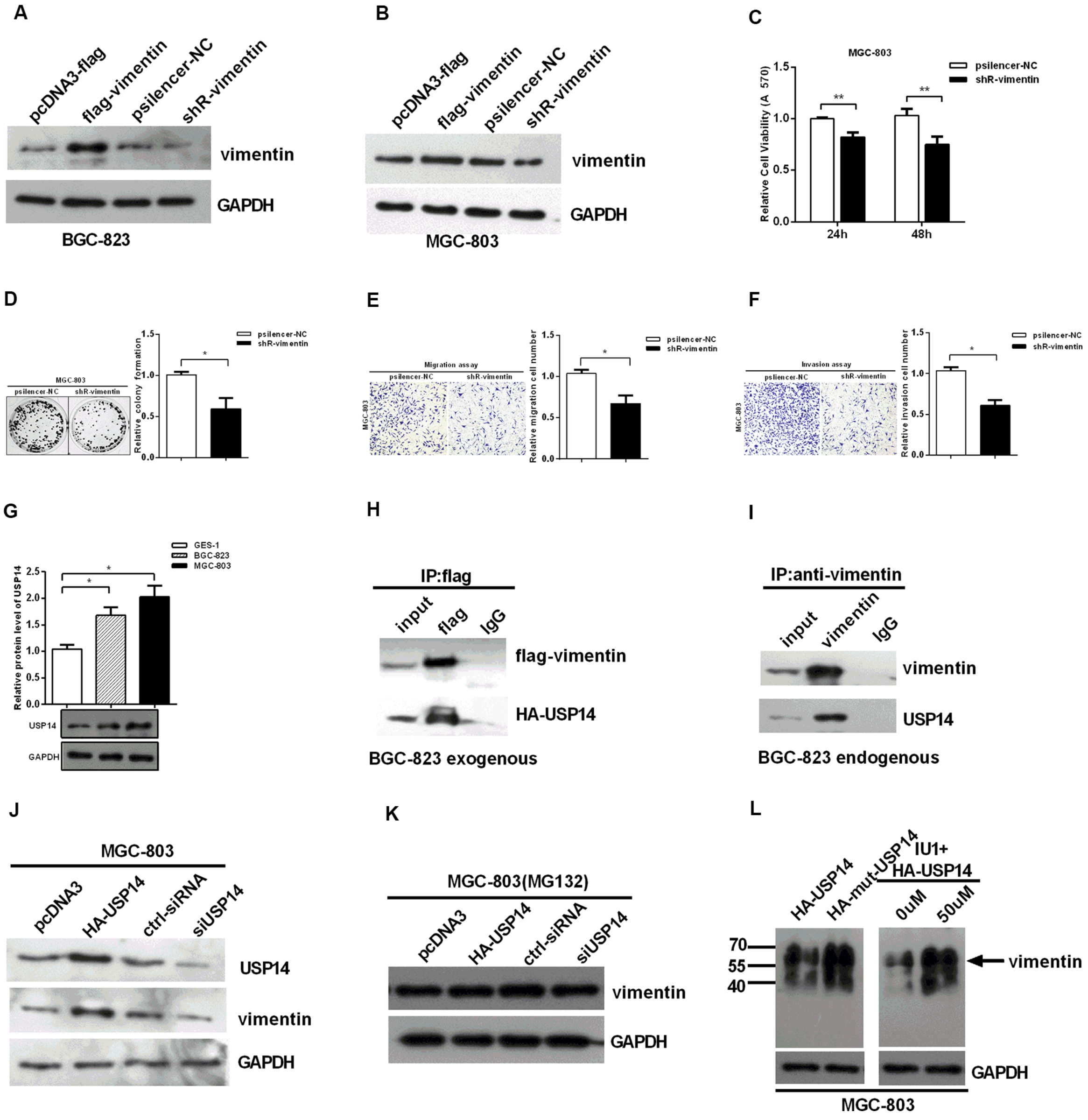

Supplementary Figure S1: The expression of vimentin via ubiquitin by USP14 influences the aggressiveness of gastric cancer cells. (**A** and **B**) Verification of the effectiveness of flag-vimentin and shR-vimentin plasmid in BGC-823 and MGC-803 cells by western blotting. (**C–F**) MTT, colony formation, migration and invasion assays performed in MGC-803 cells transfected with psilencer-NC and shR-vimentin. (**G**) Protein expression levels of USP14 in GC cell lines and GES-1 cell line. (**H** and **I**) Exogenous and endogenous Co-IP experiment exploring the interaction between USP14 and vimentin in BGC-823 cells. (**J** and **K**) Western blot analysis of the impact of USP14 on vimentin 48h after transfection and the sensitivity of vimentin to MG132 in BGC-823. (**L**) The impact of HA-mut-USP14 and USP14 inhibitor (IU1) on vimentin ubiquitin level in MGC-803. The data are presented as the mean ± S.D. $n = 3$, $^*P < 0.05$, $^{**}P < 0.01$ compared with the control group.

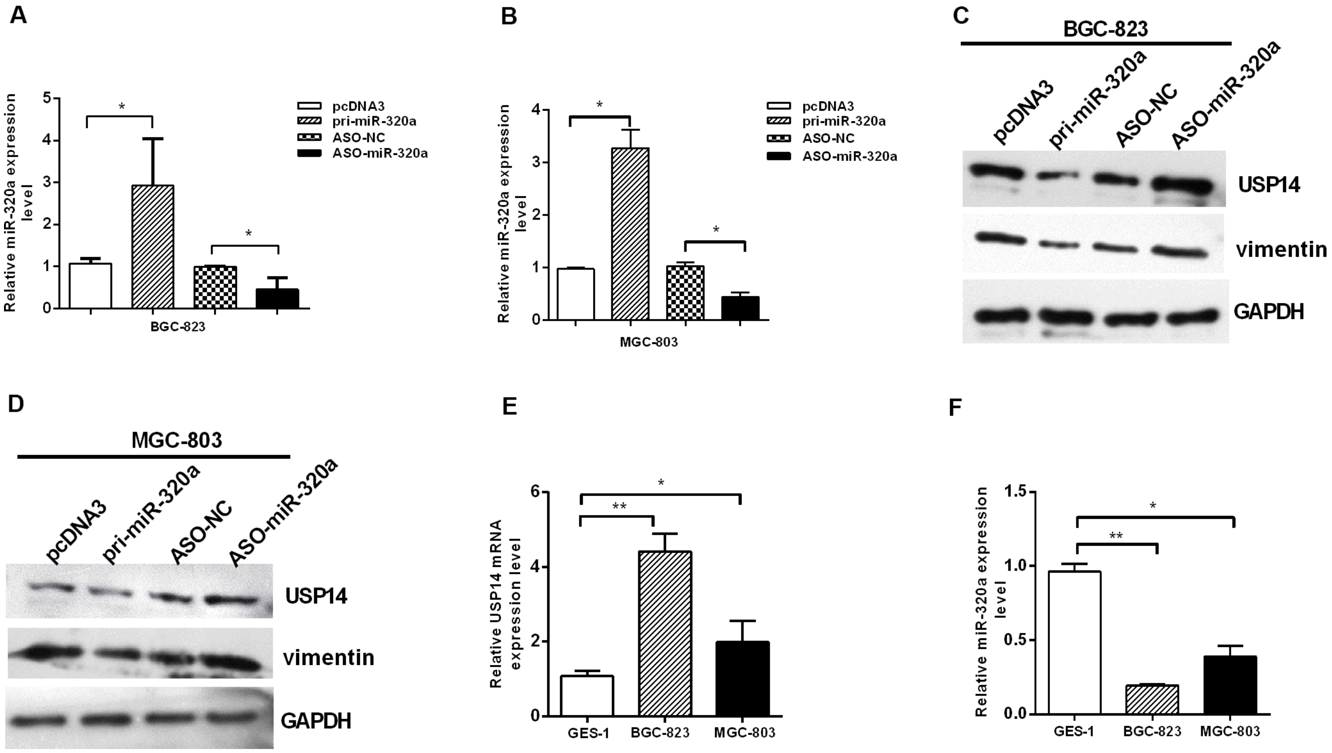

Supplementary Figure S2: The expression of USP14 and vimentin are suppressed by miR-320a in GC cells. (**A** and **B**) The transfection efficiencies of pi-miR-320a and ASO-miR-320a were evaluated by RT-qPCR. (**C** and **D**) Protein expression levels of USP14 and vimentin were measured by western blotting after transfection with pri-miR-320a and ASO-miR-320a. (**E** and **F**) The expression levels of USP14 and miR-320a in GES-1, BGC-823 and MGC-803 cells analyzed by RT-qPCR. The data are presented as the mean ± S.D. $n = 3$, $^{*}P < 0.05$, $^{**}P < 0.01$ compared with the control group.

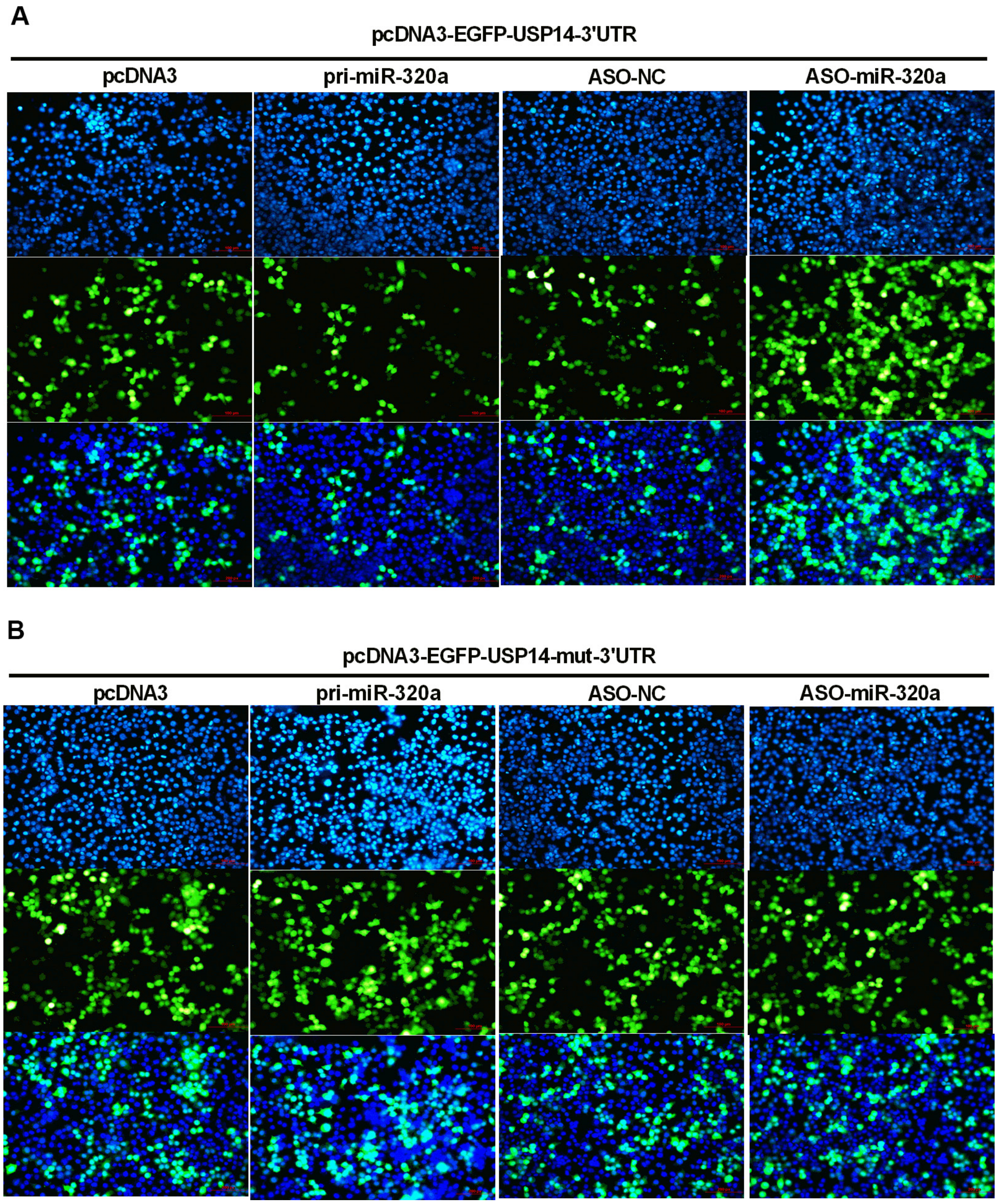

Supplementary Figure S3: Fluorescence image of EGFP reporters assays for USP14. (**A** and **B**) The representative GFP fluorescence imaging for Figure 4B.

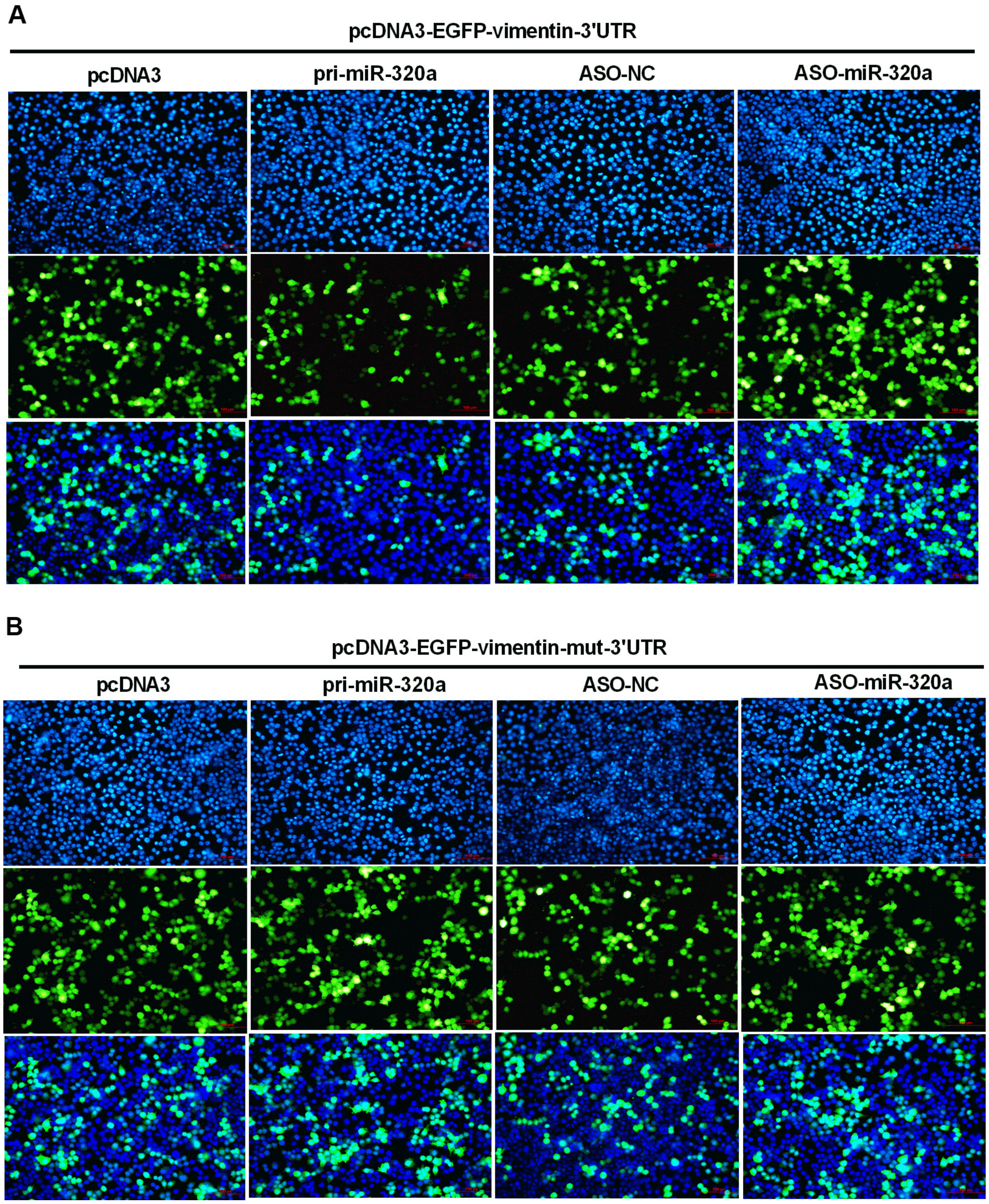

Supplementary Figure S4: Fluorescence image of EGFP reporters assays for vimentin. (**A** and **B**) The representative GFP fluorescence imaging for Figure 4C.
